# The Relationship Between Engagement in Online Support Groups and Social Isolation Among Military Caregivers: Longitudinal Questionnaire Study

**DOI:** 10.2196/16423

**Published:** 2020-04-23

**Authors:** Thomas Trail, Esther Friedman, Carolyn M Rutter, Terri Tanielian

**Affiliations:** 1 RAND Corporation Arlington, VA United States; 2 RAND Corporation Santa Monica, CA United States

**Keywords:** caregivers, family caregivers, social isolation, loneliness, depression, social support, online intervention, self-help groups, veterans health

## Abstract

**Background:**

There is a lack of research on the effectiveness of online peer support groups for reducing social isolation and depressive symptoms among caregivers, and previous research has mixed results.

**Objective:**

This study aimed to test whether military caregivers who joined a new online peer support community or engaged with an existing online community experienced decreased perceived social isolation and improved depressive symptoms over 6 months.

**Methods:**

We conducted a longitudinal study of 212 military caregivers who had newly joined an online community and those who were members of other military caregiver groups. Multiple indicators of perceived social isolation and depressive symptoms were assessed at baseline and at 3 and 6 months.

**Results:**

Compared with caregivers in the comparison group, caregivers who joined the new group experienced less perceived social isolation at 3 months (eg, number of caregivers in social network [unstandardized regression coefficients] *b*=0.49, SE 0.19, 95% CI 0.87 to 0.02), but this effect did not persist at 6 months. Those who engaged more with new or existing groups experienced less perceived social isolation over time (eg, number of caregivers in social network *b*=0.18, SE 0.06, 95% CI 0.02 to 0.27), and this relationship was mediated by increased interactions with other military caregivers (95% CI 0.0046 to 0.0961). Engagement with an online group was not associated with improvements in depressive symptoms.

**Conclusions:**

Online communities might help reduce social isolation when members engage with the group, but more intensive treatment is needed to improve depressive symptoms.

## Introduction

### Background

Serving as a caregiver to a relative or friend who is ill or wounded can be an isolating experience. Time spent caregiving can be significant [1] and may make it difficult to engage in social activities outside the home [2,3]. This is particularly true of those caring for ill or wounded service members or veterans: more than 70% of these caregivers reported feeling isolated, and approximately half of the caregivers reported hesitating to take their care recipient outside the home for fear of what might happen, especially when their care recipient experienced posttraumatic stress disorder (PTSD), anxiety, or depression [4]. These caregivers report experiencing a lack of connection to both the military and civilian community [5] and report wanting a forum to seek out social support and reduce social isolation [6]. An estimated 5.5 million people in the United States serve as informal military caregivers—family and friends who provide primarily unpaid or informal care to ill or wounded service members or veterans—and those caring for veterans of the post-9/11 conflicts are potentially facing years of caring for a young veteran with a variety of physical and mental health problems [7].

Support groups have sprung up online to provide military caregivers with an opportunity to interact with their peers and receive support and guidance (eg, the Military Veteran Caregiver Network [MVCN], Hidden Heroes, and Facebook groups). However, it is unclear how well these online groups function in terms of reducing social isolation and improving mental health among military caregivers. This study explored this question by focusing on members of a new online community for military caregivers compared with caregivers who were members of existing online support groups.

### Social Isolation and Psychological Health

Extensive research and theory suggest that people have a need to form meaningful social connections with others—that they have a need to belong [8]—so much so that when those connections are broken or nonexistent, people feel a sense of grief [8,9]. We define social isolation as a tangible or perceived deficit in social connections with other people. A tangible deficit in social connections is manifested in the structure or characteristics of an individual’s social network of connections [10]. A perceived deficit in social connections is manifested in feelings of loneliness [10] or a lack of perceived connection to others sharing similar group membership [11-13]. A connection to others can be made in person, over the phone, or online, and the connection can be with friends, family members, or relative strangers who share a common group with the individual.

Research that examines tangible deficits in social connections considers someone to be socially isolated if they live alone, have few people in their social network, or have infrequent contact with others [10]. A recent review found that people with larger and more diverse social networks (eg, networks of friends outside of their family) have lower levels of depression [14].

People can also be socially isolated if they perceive that they have a deficit in social connections with others when they feel lonely [10,15]. Feelings of loneliness are associated with the quality, rather than the quantity, of relationships; people can feel alone even when they are surrounded by others with whom they often interact [10]. The number of social ties and feelings of loneliness are not highly correlated [16,17] and often have independent effects on mental health outcomes [18]. Numerous studies have found an association between loneliness and depression, independent of social network ties [10,18].

Finally, social connections cannot only be fulfilled through tangible or perceived connections with individuals but also through perceived connections with a group [13]. Factor analyses of the University of California, Los Angeles (UCLA) Loneliness Scale have revealed that the lack of perceived connections to a group is a distinct form of social isolation [11,19]. The lack of perceived connection to a group, also called collective connectedness, is a relatively unexplored concept in the social isolation literature [19], although some previous research has considered deficits in social identity with a group as a form of social isolation [20,21]. Greater identification with a group is typically associated with better psychological health [22], including decreased depressive symptoms [23]. Thus, greater collective connectedness with a *group* of people who share similar experiences or identities (eg, as military caregivers) could represent a decrease in social isolation and may potentially foster psychological well-being.

### Online Peer Support Communities

Online forums have become popular in recent years as a way to allow peers in similar circumstances or dealing with common hardships to interact with one another, form personal bonds, and provide support to one another. One advantage of these online communities is the ability of members to elicit support from others who are dealing with similar issues but who are geographically dispersed [24,25]. Another advantage is accessibility, as users can visit online communities at their convenience. Much of a caregiver’s time is occupied with caring for a friend or loved one, and caregivers may have difficulty finding someone to provide care in their absence. Accordingly, participation in in-person support groups that require caregivers to leave their home for an hour or more at a time may be impractical. Thus, providing caregivers with access to peers via online communities has the potential to positively affect a variety of outcomes, including reducing social isolation and symptoms of depression.

However, there is a lack of research on the effectiveness of online peer support groups, and previous research has shown mixed results for improving depressive symptoms [26-30]. One review of many different types of support interventions specifically for caregivers (eg, group and individual, online, and in person) found no significant changes in quality of life, caregiver burden, depressive symptoms, or other outcomes [28]. A recent systematic review of online support interventions for caregivers found that those incorporating both peer support and professional help were most effective in improving caregiver mental health [29]. In general, although online peer support groups for caregivers of people with Alzheimer disease have shown some promise [30,31], little is known about the effectiveness of online peer support interventions for caregivers of people with mental health conditions such as PTSD [32].

Furthermore, some research suggests that active engagement with the online community (eg, reading, posting, and responding to posts to the website) may be a necessary element of the success of online support communities, but other research suggests that more passive engagement is sufficient (eg, simply visiting a site). For example, a recent study of a support group for patients with anxiety and depression found that only the most actively engaged participants experienced improvements in anxiety compared with participants who did not visit the community at all [33]. However, several studies have found that just visiting the community but not posting (ie, *lurking*) is associated with similar improvements in social outcomes compared with those who post to a community website [34,35]. To our knowledge, the role of engagement in online peer support groups among caregiver population has not been investigated in prior research. This study explored the role of engagement in the online community in decreasing social isolation and improving depressive symptoms. We examined both passive (ie, visiting the community website and spending more time on the site) and active (ie, posting to the online forum) engagement with the community.

### Military Veteran Caregiver Network

Military caregivers may especially need to connect with others who face similar challenges related to injuries and trauma experienced by service members and seek specific knowledge needed to negotiate veteran health care benefits. Thus, several online communities have been established to connect military caregivers with one another. One of these, MVCN, was established in 2016 to “provide military and veteran caregivers with peer support to reduce their isolation and increase their sense of connectedness, engagement, hopefulness, wellness as well as their knowledge and skills” [36]. MVCN allows members to post and read comments or questions to a community forum moderated by program staff; join groups organized around specific topics; exchange information about relevant resources through direct messaging; and attend webchats, webinars, and monthly question and answer calls about featured topics of interest to military caregivers. At the time of this study, web-based activities were moderated by trained paid staff who were also peer military caregivers, and there were around 1200 military caregiver members. According to interviews with MVCN staff, trained moderators and well-organized content are the features that distinguish MVCN from similar online support groups. Vaughan et al [37] give a more complete analysis of the experiences of MVCN participants.

### This Study

This study investigates whether joining an online support community helps reduce social isolation and improves depressive symptoms among caregivers. Joining an online community should increase interactions with their military caregiver peers, which should lead to improvements across several other measures of social isolation. Improvements in social isolation should lead to improvements in depressive symptoms.

To assess changes over time among new MVCN members, control for events outside of the study context (eg, changes in policy that would affect all military caregivers) and also control for systematic effects of participating in the study (eg, natural changes in ratings over time), we compared changes over time among new members of MVCN to a comparison group. We chose the comparison group to match the MVCN group along several dimensions. We wanted comparison group members to be similar to the members in the MVCN group, in that both groups comprised caregivers of ill or wounded service members or veterans. In addition, according to interviews with MVCN staff, most members of MVCN were joined through their association with other military caregiver organizations (eg, Wounded Warrior Project). Thus, we recruited caregivers for the comparison group who were members of other military caregiver organizations in an online presence but had not yet been recruited for membership in MVCN (eg, Hidden Heroes and Operation Family Caregiver). This provided a comparison group of military caregivers who were similarly involved in military caregiver issues and who were interested and willing to become a member of an online military caregiver group.

To test whether engagement in online support communities was associated with improved outcomes over time, we conducted a longitudinal study that included military caregivers who had newly joined the MVCN online community (the MVCN group) and military caregivers who had not joined MVCN but were members of other military caregiver groups (the comparison group). Study participants were assessed on multiple indicators of social isolation along with depressive symptoms at baseline and at 3 and 6 months following baseline. Data from the baseline survey were analyzed in a previous study describing the characteristics of military caregivers who join online support communities [38]. Data from the baseline and 6-month surveys, along with information from focus groups, were examined in a previous study describing the experiences of MVCN group members [37]. We sought to answer three research questions and offer tentative hypotheses for each question. However, the study is ultimately more descriptive than focused on hypothesis testing, and results were interpreted holistically across findings rather than based on individual tests of statistical significance.

#### Research Question 1: Does Joining a New Online Community Increase Connections With Other Military Caregivers and Decrease Social Isolation and Depressive Symptoms Over Time?

We hypothesized that those newly joining an online community (MVCN) would experience a greater increase in connections with other military caregivers, increase in perceived collective connectedness, decrease in perceived social isolation (ie, loneliness), and decrease in depressive symptoms over time relative to a comparison group.

#### Research Question 2: Does Engagement With Online Military Caregiver Communities, Including the Military Veteran Caregiver Network, Decrease Social Isolation and Depressive Symptoms Over Time?

On the basis of prior research detailing the relationship between social isolation and depressive symptoms, we hypothesized that engagement with an online community would function as a *dose* effect, with greater engagement leading to improvements in social isolation and depressive symptoms over time. As other online communities offer similar opportunities as MVCN for engagement with other military caregivers, we expected that the effect of engagement would be similar for both groups.

#### Research Question 3: Are the Significant Relationships Between Engagement With Online Military Caregiver Communities and Outcomes Mediated by Increased Interactions With Peers?

Although some research suggests that participating in online peer support groups can lead to improved social support and decreased depression, the role of online interactions with peers in decreasing social isolation is unclear. It is possible that online interactions are not perceived as equivalent to in-person interactions, so online interactions with caregivers may not be perceived as increasing one’s social network or be related to feelings of loneliness or collective connectedness. In addition, people can feel lonely even while interacting with others online or in person, so increased interactions with other caregivers may not mediate decreased feelings of loneliness over time. Furthermore, what does it mean to *interact* with others online? Does viewing others’ posts and replies in an online forum constitute interacting with those people? Is it roughly equivalent to being with a group of peers who are holding a conversation, but not contributing to the conversation oneself? Given our theoretical model, just being in the group but not contributing should be enough to decrease feelings of social isolation, so perhaps *perceived interactions* is a more appropriate term for this phenomenon. Indeed, research on social media use has provided mixed results as to whether engaging in online social groups decreases or actually *increases* social isolation and loneliness [39]. We expected that significant relationships between passive and active engagement with online military caregiver communities and social isolation or depressive symptoms would be mediated by increased perceived interactions with other military caregivers. From a theoretical perspective, we considered perceived interaction with peers as the mechanism that accounts for the relationship between engagement and social isolation or depressive symptoms. Therefore, where there are significant relationships between engagement and interactions with peers, we tested a mediation model of the indirect effect of engagement on social isolation or depressive symptoms through the relationship with interaction with peers. A significant mediation effect would provide additional evidence that the benefits of online communities are because of the increase in participants’ perceived ability to connect with their peers.

## Methods

### Participants

To be eligible for this study, individuals had to be adult (>18 years) military caregivers, defined here as someone who provides unpaid care and assistance for or manages the care of, a current or former member of the US military, National Guard, or Reserves who has an illness, injury, or condition for which they require outside support.

Participants in the MVCN group were recruited when they joined MVCN, and all MVCN applicants were verified to be military caregivers by virtue of their membership in other military caregiver organizations or via staff review of applicants’ documentation. New members received an email welcoming them to the group, which included text explaining the study and inviting them to participate, followed by a link to a screener survey. Of those caregivers who joined MVCN during the study recruitment period (September 2016 to February 2017), 62.0% (323/521) took the screener survey. The comparison group participants were recruited based on their membership in military caregiver organizations other than MVCN and included Hidden Heroes, Operation Family Caregiver at the Rosalynn Carter Institute for Caregiving, the Caregiving Action Network, Blue Star Families, and the American Legion Auxiliary. According to participants, most of the organizations that comparison group members belonged to offered similar activities as MVCN, including a library of information and resources, webinars, interest groups, webchats, the availability of peer mentors, and the ability to serve as peer moderators for the group. Between October 2016 and April 2017, potential comparison group participants either received an email from their member organization recruiting them for the study or the study invitation was posted on a restricted-access Facebook page administered by the group. Other than text referencing the member organization, the text of recruiting materials for comparison group participants was identical.

The screener survey included questions verifying that the participant was an unpaid caregiver for an ill or wounded service member or veteran and for those recruited for the comparison group, whether they were already a member of MVCN. Participants who successfully screened into the study then read the consent form to inform them of the purpose of the study and the survey methodology. If they indicated consent by clicking the *yes* box agreeing to participate in the study, they entered their email address and were immediately sent an email that included an individualized link to the baseline survey. All procedures were conducted in compliance with the RAND Corporation’s Human Subjects Protection Committee. Completion of the baseline survey was required for continued participation in the study. Owing to the study design, the comparison group participants were not restricted from joining MVCN during the study. As one goal of the study was to examine new members of online groups, and all participants completed the same baseline survey, a decision was made a priori to include those caregivers who were initially in the comparison group but joined MVCN between the baseline and 3-month surveys in the MVCN group (n=13, total). We asked these new members when they had joined MVCN. Of those who completed all 3 survey waves and were included in the current analysis (n=10), only 1 participant had joined the month before the survey; 1 participant had joined between 1 and 2 months before the survey, and 8 participants had joined between 2 and 3 months before the 3-month survey. This suggests that the majority of those who joined MVCN between baseline and the 3-month survey had similar exposure to MVCN as those who joined at baseline. An additional 9 comparison group participants joined MVCN between the 3- and 6-month surveys. As we were comparing changes from baseline to 3 and 6 months, those 9 participants were analyzed as members of the comparison group. Importantly, as MVCN is a closed group, only those who officially joined MVCN had access to the MVCN online forum and other resources provided to MVCN members. Those comparison group members who did not join MVCN could not access these resources.

Only participants who completed all 3 waves of surveys were included in the final analytic sample (212/345, 61% who completed the baseline assessment). We had powered the study to detect an average difference in depressive symptom score changes from baseline to 6 months of 1.25 points between the MVCN and comparison groups, assuming a correlation of 0.50 between measurements. This suggested a final sample of 58 participants per group. A post hoc power analysis using the smaller of the two groups (n=44) suggested an achieved power of 0.80 to detect a small effect between groups over time (ie, where regression partial η^2^=0.02). Participants were given a US $10 electronic gift card for completing the baseline survey, a US $10 gift card for completing the 3-month survey, and a US $20 gift card for completing the 6-month survey.

### Measures

Participants completed the following measures at baseline, 3 months, and 6 months.

#### Measure of Interactions With Peers

We assessed participants’ frequency of interacting with other military caregivers using a single item constructed for this study: “How often do you interact with other military caregivers, either in person or online (for example, texting, messaging, responding to social media posts)?” Response options included *I have never interacted with other military caregivers*, *once or twice a year or less, every few months, once a month, two or three times a month, once a week, a few times a week,* or *daily*, coded 0 to 7, respectively.

#### Social Isolation Measures

We assessed the number of military caregivers in the participant’s social network using a measure that has been shown to be associated with psychological distress in past research [40]. Participants were asked to “Think of all the people you know, who know you, and with whom you have had regular contact in the past 6 months. This contact could be face-to-face, by phone or mail, or on the internet” and to rate the number of people they know across several different categories, including “People who are also caregivers for service members or veterans.” Response categories included *none*, *1-2*, *3-4*, *5-10*, *11-20*, or *21 or more*, coded 1 to 6, respectively.

Participants’ level of collective connectedness as a military caregiver was assessed using a 3-item measure of group identity centrality [41]. Identity centrality assesses the salience of group membership in one’s life and the extent to which group membership is a core part of one’s identity [41,42] as well as their sense of belongingness and attachment to other group members (ie, their social connectedness to other group members [20,21]). Participants were asked to rate their agreement with the following 3 items using a 5-point scale (from *strongly disagree* to *strongly agree*): “In general, being a military caregiver is an important part of my self-image,” “I have a strong sense of belonging to the military caregiver community,” “I have a strong attachment to other military caregivers” (Cronbach alpha=.86 at baseline).

Loneliness was measured using the 3-item short scale for measuring loneliness [17], which has been used in the National Social Life, Health, and Aging Project [43]. Participants were asked to rate how often they feel they *lack companionship*, *feel left out*, and *feel isolated* using a 4-point scale (*hardly ever*, *some of the time*, *most of the time*, or *always*). The average of the 3 items was computed to form the loneliness scale (Cronbach alpha=.89 at baseline). This measure was chosen because of its broad applicability to online relationships, whereas other loneliness measures have items that are more suitable for assessing in-person or family relationships. For example, the UCLA Loneliness Scale refers to *being alone* and feeling isolated from *those around you* [11], which could be interpreted as applying to in-person relationships only. The Social and Emotional Loneliness Scale for Adults assesses relationships with friends, family, and romantic partners [44], which are not relevant categories for online communities.

#### Measure of Depressive Symptoms

Depressive symptom severity was measured using the 8-item version of the Patient Health Questionnaire (PHQ-8), which is a clinically validated measure of depressive symptoms based on the Diagnostic and Statistical Manual of Mental Disorders, Fourth Edition criteria for depressive disorders [45,46]. Participants rated the extent to which they had been bothered by 8 symptoms of depression in the past 2 weeks (eg, *having little interest or pleasure in doing things*) using a 4-point scale, ranging from 0 (*not at all*) to 3 (*nearly every day*). Scores were summed to create an index of depressive symptoms ranging from 0 to 24 (Cronbach alpha=.90 at baseline).

#### Engagement in Online Community Measures

In the 3-month survey, participants who were members of MVCN were asked a series of questions about their engagement with the MVCN community. Participants who were not members of MVCN (comparison group participants) were asked to name the online community of caregivers, which they *engage with the most (spend time at the website, post comments, attend webinars, etc).* The same questions assessing engagement were asked of the group named by each participant.

Passive engagement with the online community was assessed by measuring the frequency of visits to the website and the time spent on the website. The frequency of visits to the community website was measured via 1 item: *Since joining [group], how often have you visited the [group] website?* Where *group* was either *MVCN network* or the online group with which comparison group participants engaged the most. Response options and numeric codes were *every day* (6), *almost every day* (5), *two or three times a week* (4)*,* *once a week* (3), *two or three times a month* (2), *once a month or less* (1), or *I have not visited the website for [group] since joining this group* (0). The length of time spent on the site was measured by asking, “When you visit, how much time do you usually spend on the [*group*] website?” Where *group* was either *MVCN network* or the online group with which comparison group participants engaged the most. Response options and numeric codes were *less than 10 min* (1), *between 10 and 20 min* (2), *20 to 30 min* (3), *30 min to an hour* (4), or *an hour or more* (5).

Active engagement with the online community via posts to the online forum was assessed by asking, “Have you posted any comments, questions, or links to the [*group*]? Where *group* was either *MVCN network* or the online group with which comparison group participants engaged the most. Their responses were coded 1 if *yes* and 0 if *no*.

#### Covariate Measures

We examined several potential covariates for inclusion in the analysis, including caregiver age, gender, race, level of education, household income, hours spent providing care in a typical week, caregiver’s relationship with the care recipient, whether the care recipient served during the pre- or post-9/11 period, the number of activities of daily living and instrumental activities of daily living that the care recipient needed help performing, membership in other structured support groups, veteran’s age, years spent caring for the care recipient, and whether the caregiver lived in a metropolitan area. For each of these, we examined whether the variable was significantly related to MVCN membership, any of the engagement variables, or attrition from the study at 3 and 6 months. *t* tests, chi-square tests, and regression analyses were used to test for differences, as appropriate. A conservative criterion of *P*<.10 was used in these analyses. We found that MVCN and comparison group participants differed in veteran care recipient age (mean 40.2, SD 10.6 years for MVCN and mean 45.4, SD 18.3 years for comparison care recipients). No other differences emerged between the MVCN and comparison group participants. Being a member of another online or in-person support group was associated with more visits to the MVCN or comparison group community website. In addition, males spent more time on the respective community website, as did those caregivers who helped their care recipients with more tasks and those who lived outside of metropolitan areas. Those caring for older veterans, living outside of metropolitan areas, and who were members of another support group were more likely to discontinue participation in the study over time. Thus, we controlled for these 3 variables plus caregiver gender, membership in other online support groups, and number of caregiver tasks in all analyses. Note that all analyses reported below replicate when these covariates were not included in the models. Finally, to distinguish the effects of visits to the website from time spent on the site and posts to the forum, we controlled for any visits to the website (yes/no) in the analysis of these variables.

### Analytic Approach

Our analytic approach focused on assessing changes in outcomes from the baseline assessment to the 3- and 6-month assessments. We conducted two separate sets of models using multiple regression: one set of models regressed 3-month outcomes on baseline predictors controlling for baseline levels of outcomes and covariates, and the other set regressed 6-month outcomes on baseline predictors controlling for baseline levels of outcomes and covariates. For each set of models, to answer research question 1, we first analyzed changes from baseline for new members of an online community (MVCN) in comparison with those who were already members of one or more online communities other than MVCN (comparison group). To answer research question 2, we used cross-lagged models to examine changes in outcomes from baseline to 6 months as a function of engagement with the online community at 3 months (ie, we added a measure of engagement at 3 months to the 6-month models examined in research question 1). We also assessed whether this relationship differed for MVCN and comparison participants, based on an interaction between MVCN membership (vs comparison group) and 3-month engagement. Cross-lagged models allow for explanatory predictions of present outcomes based on past behaviors and are thus more likely to reflect causal effects than are regressions using cross-sectional measures. Although we conducted statistical tests for each model and included the associated *P* values for the tests, we provided 95% CIs for each result and interpreted our findings based on these CIs and the pattern of results across findings, rather than based on the statistical significance of any one test.

Finally, for research question 3, we conducted mediational analyses for models where engagement significantly predicted both interactions with other military caregivers and one or more outcomes. Engagement at 3 months was entered as the predictor variable, with changes in the frequency of interactions with other military caregivers from baseline to 6 months serving as the mediator. A difference score was calculated for changes in interactions from baseline to 6 months, and we included the baseline measure of interactions as a covariate to control for the possibility that the extent of changes in interactions was driven by baseline levels of interactions. Outcomes were measured at month 6, and changes in outcomes were assessed by including baseline levels of each outcome in the models. Mediational analyses were conducted in SAS 9.3 (SAS Institute Inc) using the INDIRECT macro, which uses asymptotic bootstrapping calculations to test for the significance of indirect effects [47]. The INDIRECT macro generated 95% bias-corrected SEs and CIs using 5000 bootstrapped samples, and mediation models were considered significant when the CI for the estimation of the indirect effect did not contain 0.

## Results

### Participant Characteristics

Of the 212 participants who completed all 3 waves of the study, 199 (93.9%) were female, 166 (78.3%) were non-Hispanic white, 23 (10.8%) were Hispanic, and 23 (10.8%) were in other racial/ethnic groups. The majority of caregivers were spouses or partners of the veteran for whom they provided care (189/212, 89.2%), and the remaining caregivers were mostly other family members (1 participant was a friend or neighbor of the veteran). Most caregivers were under the age of 40 years (124/212, 58.5%), with an additional 74 (34.9%) aged between 40 and 59 years. The care recipients were mostly male (188/212, 88.7%), with 125 (59.0%) serving during the post-9/11 period. Almost all veterans had been diagnosed with one or more physical health conditions (203/212, 95.8%), with 182 (85.8%) diagnosed with more than one physical health problem. In addition, 87.7% (186/211) of veterans had been diagnosed with one or more psychological health conditions, with 174 (82.1%) veterans diagnosed with PTSD. We compared those who completed all 3 waves of the survey with those who did not complete all surveys on baseline-level demographic characteristics and all variables included in this study and found no significant differences between groups. In comparison with the overall population of military caregivers studied by Ramchand et al [7], participants in this study were more likely to be female, non-Hispanic white, married to the care recipient, and caring for a veteran with one or more psychological health conditions, particularly PTSD. It is unclear whether these differences reflect the population of members of online communities or only those who participated in this study.

Means and SDs for outcome variables (at baseline) and engagement variables (at 3 months) are shown in Table 1. Mean scores for variables at baseline were generally near the midpoint of the range of possible scores. Missing data were uniformly low (8/212 or less). Using a cutoff score of 10 or higher to indicate probable major depressive disorder on the PHQ-8 [45,46], 59.4% (126/212) of participants screened positive for probable depression at baseline, which is a higher proportion than that found in the general military caregiver population [7]. One statistically significant difference between MVCN and comparison group participants emerged on outcomes measured at baseline: MVCN participants reported more frequent interactions with other military caregivers than did comparison group members (2-tailed t_210_=2.08; *P*=.04).

Among engagement variables measured at 3 months, 25.8% (42/163) of MVCN and 23.3% (10/43) of comparison group participants indicated that they had not visited the community website since baseline (χ^2^_1_=0.1; *P*=.74), and the comparison group participants visited the website more often than MVCN participants (2-tailed t_204_=3.16; *P*=.002). Among those who visited their community’s website, the average time spent on the website was around 10 and 20 min (coded as *2*). MVCN group participants were also less likely to have posted to the site than were comparison participants (χ^2^_1_=26.1; *P*<.001).

**Table 1:** Means and SDs for outcome and engagement variables by group.

Variable	Range	MVCN^a^				Comparison group		
		Baseline	3 months	6 months	Baseline		3 months	6 months
Frequency of interacting with military caregivers, mean (SD)^b^	0-7	5.08 (2.47)	5.17 (2.41)	5.17 (2.25)	4.20 (2.61)		4.02 (2.50)	4.21 (2.40)
Military caregivers in network, mean (SD)^b^	1-6	2.48 (1.50)	2.51 (1.47)	2.50 (1.49)	2.32 (1.49)		1.95 (1.08)	2.09 (1.17)
Collective connectedness with military caregivers, mean (SD)^b^	1-5	2.98 (1.20)	3.02 (1.18)	3.05 (1.20)	2.86 (1.07)		2.72 (1.00)	2.61 (1.18)
Loneliness, mean (SD)^b^	3-12	8.01 (2.36)	7.70 (2.51)	7.90 (2.33)	7.36 (2.62)		7.89 (2.39)	7.21 (2.64)
Depression severity PHQ-8^c^, mean (SD)^b^	0-24	11.35 (6.66)	10.29 (6.51)	10.25 (6.29)	9.89 (5.84)		10.02 (6.10)	9.21 (6.68)
Frequency of visiting online community, mean (SD)^b^	0-6	N/A^d^	1.41 (1.32)	N/A	N/A		2.23 (2.11)	N/A
Average amount of time on site per visit, mean (SD)^b^	0-5	N/A	1.61 (1.30)	N/A	N/A		1.33 (1.15)	N/A
Posted to site, n (%)	N/A	N/A	27 (16.6)	N/A	N/A		24 (55.8)	N/A

^a^MVCN: Military Veteran Caregiver Network.

^b^Means and SDs are unadjusted for other variables in the regression models.

^c^PHQ-8: 8-item patient health questionnaire.

^d^N/A: not applicable.

### Results for Research Question 1: Does Joining a New Online Community Increase Connections With Other Military Caregivers and Decrease Social Isolation and Depressive Symptoms Over Time?

Comparing social isolation outcomes for MVCN and comparison group members at 3 months, controlling for baseline levels of outcomes and covariates, yielded two statistically significant findings: compared with comparison group members, MVCN members reported a higher number of military caregivers in their social network, and MVCN members reported feeling less lonely (Table 2). These differences did not persist at 6 months. The only significant difference among social isolation measures at 6 months was that MVCN members experienced relatively greater collective connectedness with other military caregivers compared with comparison group members (Table 2).

**Table 2:** Regression results predicting difference from baseline to 3-month outcomes and 6-month outcomes by membership in Military Veteran Caregiver Network vs comparison group (N=212).

Outcome	MVCN^a^ vs comparison group^b^						
	3-month outcomes				6-month outcomes		
	*b* (95% CI)	SE	ΔR^2^	*b* (95% CI)		SE	ΔR^2^
Frequency of interacting with other military caregivers	0.49 (−0.12 to 0.97)	0.19	0.005	0.22 (−0.33 to 0.77)		0.28	0.001
Number of military caregivers in network	0.49^c^ (0.11 to 0.87)	0.19	0.020	0.33 (0.04 to 0.95)		0.21	0.009
Collective connectedness with military caregivers	0.15 (−0.12 to 0.41)	0.14	0.003	0.39^d^ (0.10 to 0.69)		0.15	0.017
Loneliness	−0.67^c^ (−1.33 to −0.01)	0.34	0.012	0.16 (−0.57 to 0.89)		0.37	0.001
Depression severity PHQ-8^e^ score	−0.93 (−2.54 to 0.69)	0.82	0.003	0.08 (−1.78 to 1.94)		0.94	0.000

^a^MVCN: Military Veteran Caregiver Network.

^b^Models controlled for caregiver gender, membership in any other support group, number of caregiving tasks, veteran age, metropolitan residence, and baseline levels of the outcome. Larger numbers indicate an increase in MVCN participants over time relative to the comparison group participants.

^c^*P*<.05.

^d^*P*<.01.

^e^PHQ-8: 8-item Patient Health Questionnaire.

### Results for Research Question 2: Does Engagement With Online Military Caregiver Communities, Including Military Veteran Caregiver Network, Increase Connections With Other Military Caregivers and Decrease Social Isolation and Depressive Symptoms Over Time?

The next set of analyses examined the relationship between engagement with online caregiver communities at 3 months with changes in outcomes from baseline to 6 months. The results are shown in Table 3. The number of visits to the online community at 3 months was significantly associated with a greater increase in the frequency of interactions with other military caregivers, the number of military caregivers in one’s social network, and in collective connectedness with other military caregivers from baseline to 6 months. The average time spent on the community website at 3 months was significantly associated with a decrease in feelings of loneliness and an increase in collective connectedness from baseline to 6 months. Posting to the online community was significantly associated with an increase in the number of military caregivers in one’s social network from baseline to 6 months, but no other significant relationships with posting were observed. Finally, no significant interactions between any of the engagement variables and MVCN membership were observed.

**Table 3:** Regression results predicting baseline to 6-month changes by 3-month engagement with online community (N=212).

Outcome	Number of visits to online community^a^			Time spent in online community^a^			Post to online community forum (yes or no)^a^		
	*b* (95% CI)	SE	ΔR^2^	*b* (95% CI)	SE	ΔR^2^	*b* (95% CI)	SE	ΔR^2^
Frequency of interacting with other military caregivers	0.18^b^ (0.03 to 0.33)	0.08	0.012	0.08 (−0.17 to 0.33)	0.13	0.001	0.32 (−0.25 to 0.89)	0.29	0.003
Total number of military caregivers in network	0.18^c^ (0.02 to 0.27)	0.06	0.030	0.02 (−0.18 to 0.22)	0.10	0.000	0.61^c^ (0.18 to 1.04)	0.22	0.026
Collective connectedness with military caregivers	0.20^d^ (0.12 to 0.28)	0.04	0.057	0.15^b^ (0.01 to 0.29)	0.07	0.011	0.19 (−0.11 to 0.50)	0.15	0.004
Loneliness	−0.15 (−0.35 to 0.05)	0.10	0.008	−0.42^b^ (−0.75 to −0.08)	0.17	0.022	−0.04 (−0.81 to 0.73)	0.39	0.000
Depression severity PHQ-8^e^ score	0.23 (−0.29 to 0.74)	0.26	0.003	−0.18 (−1.05 to 0.69)	0.44	0.001	1.24 (−0.72 to 3.21)	1.00	0.005

^a^Covariates included in the models were caregiver gender, membership in any other support group, number of caregiving tasks, veteran age, metropolitan residence, membership in Military Veteran Caregiver Network, and baseline levels of the outcome. Analysis of time on site and posts to online community also included a dummy variable for any visits to the site.

^b^*P*<.05.

^c^*P*<.01.

^d^*P*<.001.

^e^PHQ-8: 8-item Patient Health Questionnaire.

### Results for Research Question 3: Are the Significant Relationships Between Engagement With Online Military Caregiver Communities and Outcomes Mediated by Increased Interactions With Peers?

The number of visits to the online community at 3 months was significantly associated with changes in the frequency of interactions with other military caregivers (the hypothesized mediator) and with the total number of military caregivers included in one’s social network and collective connectedness with other military caregivers. First, examining the number of military caregivers in one’s social network, analysis using the INDIRECT macro in SAS [47] replicated our earlier findings: number of visits at 3 months was significantly associated with an increase in social network connections from baseline to 6 months and with changes in interactions with other military caregivers. When included in the full model, an increase in interactions with other military caregivers was also significantly associated with changes in social network connections ([unstandardized regression coefficients] *b*=0.22; SE 0.05; *P*<.001), and the relationship between the number of visits and the changes in social network connections was reduced to marginal significance (*b*=0.11; SE 0.06; *P*=.051). Analyses confirmed that the mediation model was significant (95% CI 0.0046 to 0.0961).

Next, examining changes in collective connectedness with other military caregivers, analysis using the INDIRECT macro replicated our earlier findings: number of visits at 3 months was significantly associated with an increase in collective connectedness from baseline to 6 months and with changes in interactions with other military caregivers. When included in the full model, an increase in interactions with other military caregivers was also significantly associated with changes in collective connectedness (*b*=0.19; SE 0.04; *P*<.001), but the relationship between the number of visits and changes in collective connectedness remained significant (*b*=0.17; SE 0.04; *P*<.001). Analyses confirmed that the mediation model was significant (95% CI 0.0022 to 0.0719).

## Discussion

### Principal Findings and Implications

This study suggests that relative to a comparison group, military caregivers joining a new online community such as MVCN experience less social isolation and loneliness over time, that the benefits of membership in new or existing online caregiver communities are greater for those who engage more with the community, and that the relationships between engagement and social isolation are mediated by an increase in participant interactions with other military caregivers. The effects of decreased loneliness are particularly noteworthy as loneliness has been associated with an increased risk of mortality and poor mental health across a variety of indicators [10]. However, our results did not demonstrate that engagement with online communities was associated with improvements in depressive symptoms over time.

Our findings suggest that joining a new group such as MVCN may have a short-term impact on social isolation in terms of increasing the number of military caregivers in one’s social network and decreasing feelings of loneliness. These gains do not appear to continue at 6 months, possibly because new MVCN members formed new connections with peers that reached saturation at 3 months. MVCN members’ collective connectedness with other military caregivers showed an increase at 6 months, relative to comparison group members. As MVCN members were recruited right when they joined the group, the MVCN community was a new environment for them to meet and form bonds with other military caregivers. Comparison group members were recruited via groups to which they already belonged, so their ties to their existing groups were more established, and their collective connectedness with other caregivers was possibly more formed. Thus, the effects of joining MVCN likely reflect engagement in a new environment with presumably new people who share a similar identity and similar challenges. It is perhaps not surprising that those joining a new group experienced reduced social isolation—it demonstrates that the group is doing what it was primarily designed to do for military caregivers.

Joining MVCN was not associated with improvements in depressive symptoms. MVCN participants scored relatively high on the PHQ-8, with 61.1% (102/167) having a PHQ-8 score of 10 or above at baseline, suggesting probable major depressive disorder. A review of web-based interventions for caregivers revealed that these interventions show some promise for reducing depressive symptoms [48], and recent research evaluating the impact of an in-person support program for military caregivers demonstrated improvements in depressive symptoms among participants [49]. It is possible that joining a new online support group is not enough of an intervention on its own to reduce depressive symptoms, and these studies suggest that more intensive online or in-person interventions might be needed to successfully treat depression among military caregivers.

Engagement in online communities across the MVCN and comparison groups was associated with a decrease in social isolation at 6 months. Cross-lagged models predicting 6-month outcomes from engagement at 3 months revealed that more visits to the community at 3 months were associated with greater gains in the number of military caregivers in one’s social network and increased collective connectedness with other military caregivers at 6 months. Furthermore, both these relationships were mediated by an increase in participant interactions with other military caregivers, suggesting that simply visiting an online community of peers more often can increase interactions with peers, which is then associated with reduced social isolation. In addition, spending more time on the community website was associated with increased collective connectedness and decreased feelings of loneliness at 6 months. Posting to online community forums was only associated with gains in social network connections with military caregivers at 6 months. Thus, across the three indicators of engagement with an online community of peers, we found that increased engagement was associated with decreased social isolation over time, and more active engagement in the forums through posting was not associated with additional improvements in loneliness or social network size. Although engagement was not associated with a decrease in depression, our results support the theory that engaging with online social networks might help reduce social isolation, particularly when they enable users to make meaningful connections with others [39], which suggests that programs implementing online support groups should use strategies to encourage members to visit and spend time on the community website, at a minimum.

One aspect of the MVCN community forum that might have affected engagement with the group was the active role that online forum moderators play in that group. Users prefer online support groups that are facilitated by trained peer moderators that provide trustworthy information [50]. The MVCN community forum is facilitated by a team of trained peer moderators, and research using focus groups of MVCN members and initial survey data from this study found that the information provided by the website and forum moderators was trustworthy compared with other online forums [37]. It is possible that active engagement by trained peer moderators served to improve the impact of engagement with MVCN on members’ well-being. However, this research also found that participation in MVCN was generally low and mostly passive [37], which suggests that the presence of trained peer moderators could also have decreased more active engagement with the online forum (eg, participants might not respond to posts requesting information if they think that a trained moderator will respond instead). This is not necessarily a bad trade-off because the engagement of peer facilitators might make the forum more trustworthy and decrease the spread of rumors and misinformation. Additional research is needed to determine the beneficial impact that trained peer moderators might have on participants in online support groups.

### Strengths and Limitations

Although this study has several strengths, including 3 waves of data, several measures of social isolation, and the ability to conduct causal analyses using cross-lagged models, it also has several limitations. First, participants were not randomly assigned to groups, so MVCN participants could have systematically differed from comparison group participants in ways that affected our results, including their motivation or ability to engage with their online community and their proclivity for change over time. Furthermore, we did not control group membership, so comparison group participants were able to join MVCN during the study, and 10 comparison group participants included in this study had joined MVCN at the 3-month survey. We made an a priori decision to include these participants in the MVCN group because their data at 3 and 6 months would reflect similar experiences with MVCN as those who had joined MVCN at baseline. Most (8/10) of these new members had joined MVCN between 2 and 3 months before the 3-month survey. However, an additional 9 comparison group participants indicated that they had joined MVCN at the 6-month survey and were counted as comparison group participants for the purposes of this study. A post hoc analysis excluding these participants from the study did not substantially change the results, although a significant effect of the MVCN group on the number of military caregivers in one’s social network emerged at the 6-month assessment, with MVCN members reporting increased numbers of military caregivers in their social network from baseline to 6 months relative to comparison group members (*b*=0.49; SE 0.23; 2-tailed t_181_=2.15; *P*=.03; ΔR^2^=0.017). This finding suggests that the decision to keep new MVCN members at 6 months in the comparison group made our results comparing the two groups more conservative, rather than biasing the results toward significant differences. However, in combination with the lack of random assignment, the results distinguishing the MVCN participants from the comparison group participants need to be interpreted with caution. In addition, we did not control engagement in online communities, so individual differences in propensity to engage with online communities could have driven the effects reported here. Finally, engagement was self-reported, so individual biases in reporting engagement could have affected the results. Using a within-person design and examining cross-lagged effects helps correct for these biases but does not completely rule them out. Future research should randomly assign participants to groups and manipulate engagement with online communities to better assess causality.

### Conclusions

Although helping military caregivers to overcome feelings of social isolation is an important contribution of online support groups such as MVCN, our study indicates that more intensive efforts may be needed to improve depressive symptoms. This will likely include opportunities that promote treatment seeking and offer more structured peer or professional support through online [51] or in-person connections.

## Abbreviations

MVCN: Military Veteran Caregiver Network
PHQ-8: 8-item Patient Health Questionnaire
PTSD: posttraumatic stress disorder
UCLA: University of California, Los Angeles
